# The economic burden of RSV-associated illness in children aged < 5 years, South Africa 2011–2016

**DOI:** 10.1186/s12916-023-02854-2

**Published:** 2023-04-13

**Authors:** Jocelyn Moyes, Stefano Tempia, Sibongile Walaza, Meredith L. McMorrow, Florette Treurnicht, Nicole Wolter, Anne von Gottberg, Kathleen Kahn, Adam L. Cohen, Halima Dawood, Ebrahim Variava, Cheryl Cohen

**Affiliations:** 1 Centre for Respiratory Diseases and Meningitis, National Institute for Communicable Diseases of the National Health Laboratory Service, Johannesburg, South Africa; 2 School of Public Health, Faculty of Health Sciences, University of the Witwatersrand, Johannesburg, South Africa; 3 Division of Viral Diseases, Centers for Disease Control and Prevention, Atlanta, GA USA; 4 Division of Virology, Faculty of Health Sciences, National Health Laboratory Service, Charlotte Maxeke Johannesburg Academic Hospital, Johannesburg, South Africa; 5 School of Pathology, Faculty of Health Sciences, University of the Witwatersrand, Johannesburg, South Africa; 6 MRC/Wits Rural Public Health and Health Transitions Research Unit (Agincourt), School of Public Health, Faculty of Health Sciences, University of the Witwatersrand, Johannesburg, Epidemiology and Global Health Unit, Johannesburg, South Africa; 7 Division of Bacterial Diseases Division, Centers for Disease Control and Prevention, Atlanta, GA USA; 8 Department of Medicine, Greys Hospital, Pietermaritzburg, South Africa; 9 Caprisa, University of KwaZulu-Natal, Pietermaritzburg, South Africa; 10 Department of Medicine, Klerksdorp-Tshepong Hospital Complex, Klerksdorp, South Africa; 11 Department of Medicine, Faculty of Health Sciences, University of the Witwatersrand, Johannesburg, South Africa; 12 Perinatal HIV Research Unit, University of the Witwatersrand, Johannesburg, South Africa

**Keywords:** Burden, Cost, Respiratory syncytial virus, Children, Respiratory illness

## Abstract

**Background:**

Data on the economic burden of RSV-associated illness will inform decisions on the programmatic implementation of maternal vaccines and monoclonal antibodies. We estimated the cost of RSV-associated illness in fine age bands to allow more accurate cost-effectiveness models to account for a limited duration of protection conferred by short- or long-acting interventions.

**Methods:**

We conducted a costing study at sentinel sites across South Africa to estimate out-of-pocket and indirect costs for RSV-associated mild and severe illness. We collected facility-specific costs for staffing, equipment, services, diagnostic tests, and treatment. Using case-based data we calculated a patient day equivalent (PDE) for RSV-associated hospitalizations or clinic visits; the PDE was multiplied by the number of days of care to provide a case cost to the healthcare system. We estimated the costs in 3-month age intervals in children aged < 1 year and as a single group for children aged 1–4 years. We then applied our data to a modified version of the World Health Organization tool for estimating the mean annual national cost burden, including medically and non-medically attended RSV-associated illness.

**Results:**

The estimated mean annual cost of RSV-associated illness in children aged < 5 years was US dollars ($)137,204,393, of which 76% ($111,742,713) were healthcare system incurred, 6% ($8,881,612) were out-of-pocket expenses and 13% ($28,225,.801) were indirect costs. Thirty-three percent ($45,652,677/$137,204,393) of the total cost in children aged < 5 years was in the < 3-month age group, of which 52% ($71,654,002/$137,204,393) were healthcare system incurred. The costs of non-medically attended cases increased with age from $3,307,218 in the < 3-month age group to $8,603,377 in the 9–11-month age group.

**Conclusions:**

Among children < 5 years of age with RSV in South Africa, the highest cost burden was in the youngest infants; therefore, interventions against RSV targeting this age group are important to reduce the health and cost burden of RSV-associated illness.

## Background

Globally, an estimated 33 million RSV-associated lower respiratory tract infections (LRTI) and 118,000 (95% confidence interval (CI) 94,600–149,400) deaths occur annually in children under 5 years of age [1]. The only currently licensed respiratory syncytial virus (RSV)-prevention method is a short-acting monoclonal antibody (mAb) (palivizumab) injected monthly, usually recommend for high-risk infants due to cost. mAb are not widely available in many low- and middle-income settings such as South Africa. However, a number of new technologies, including maternal vaccines and longer-acting monoclonal antibodies, are being developed to prevent RSV-associated illness in children [2].

Global cost-effectiveness models based on estimates of burden in low- and middle-income countries suggest up to 1.2 million cases, 104 million hospital admissions, and 3000 deaths could be averted annually through maternal RSV vaccination, including a cost saving of 186 million US dollars ($) in healthcare costs [3]. Monoclonal antibodies strategies could prevent even more cases, with more cost savings than a maternal vaccine [3]. In a recent global analysis, cost-effectiveness was modeled in two age bands, < 1 year and < 5 years. These broad age bands do not account for the fact that mAb and maternal vaccines in trials may have a limited duration of effect (up to 6 months) within the < 1-year age band. Cost-effectiveness models may be improved by considering finer age bands in infants, specifically in infants < 6 months of age. Li et al., for their model of cost-effectiveness, list the following as important parameters to quantify for country-level cost-effectiveness models; cost of hospitalized and non-medically attended cases and mortality burden, to ensure cost-effectiveness models are accurate and specific [3].

In South Africa the burden of RSV-associated mild and severe illness is substantial. From 2012 to 2016, the mean annual number of RSV-associated severe cases in children aged < 5 years was 96,220 (95% confidence interval (CI) 66,470–132,844) with 78,571 (95% CI 56,187–105,831) severe cases in children aged < 1 year. In the same period the mean number of RSV-associated deaths in children aged < 5 years was 650 (95% CI 475–947) [4]. There were also a large number of non-medically attended cases for both severe and mild RSV-associated illness (204,080 95% CI 103,191–341,183 and 51,605 95% CI 33,739–75,306, respectively) [4]. With these detailed burden estimates, we can describe the cost burden of RSV-associated illness to inform country-level cost-effectiveness models.

We aimed to estimate the direct cost of services (healthcare system costs), direct and indirect costs to the caregiver (out-of-pocket costs and loss of income), and cost burden (total costs applied to the national burden of RSV-associated illness) associated with RSV-associated mild and severe illness in children aged < 5 years (in 3-month age groups from 0 to 11 months and then in those aged 1–4 years) in South Africa from 2011 through 2016. We also estimate the cost of illness in children who do not access healthcare for the episode (non-medically attended).

## Methods

We collected all costs in South Africa Rands (ZAR) and converted to US dollars ($) using the average monthly ZAR to $ exchange in 2014 based on the South African all-items consumer price index [5]. We used the terms healthcare provider cost to describe cost related to outpatient visits and hospitalization costs, which in our setting are costs covered by the provincial departments of health, i.e., no cost to the caregiver. These cases are referred to as medically-attended mild (outpatient) or severe cases (hospitalized). Cost assigned to the caregiver is direct cost (pre-visit/hospitalization care, medication prior to the visits, and transport) and indirect (loss of income costs) of the illness episode. We use the term non-medically attended illness costs to describe the cost to caregivers, direct and indirect of illness episodes where healthcare was not accessed.

### Study setting and study population

We conducted a costing study at sentinel surveillance sites (5 hospitals and 2 public health clinics in 4 of South Africa’s 9 provinces) that are part of a national surveillance program for pneumonia to estimate out-of-pocket and indirect costs incurred by caregivers of children with RSV-associated mild (influenza-like illness (ILI)) and severe illness (severe acute respiratory illness (SARI)) from January through December 2014 providing a baseline of costing estimates (and adjusted for consumer price index for other years). The national surveillance program for pneumonia was started in 2009 and runs at sentinel sites across the country. In 2012, we added primary healthcare clinics in the sentinel hospital catchment area. Dedicated surveillance officers enroll patients Monday through Friday and collect demographic, clinical, and treatment data. Nasopharyngeal swabs are collected from patients and tested at the National Institute for Communicable Diseases (NICD) for a number of respiratory viruses, including RSV.

We selected the children for the costing study from children enrolled in the surveillance program (outpatient and hospitalized) based on target numbers of 2 children aged < 1 year and 2 children aged 1–4 years every week for 1 year irrespective of RSV result. These children were sampled systematically; we included the first two identified children meeting the study enrolment criteria each week. Children who were enrolled in the outpatient surveillance program and then subsequently admitted to the hospital within 14 days were excluded from enrolment in the costing study to avoid overlap between mild and severe cases.

The case definition for ILI included fever and cough (duration of symptoms < 10 days). We refer to this as mild illness for ease of reading. This definition was used for children attending outpatient clinics. In addition, we used a physician diagnosis of LRTI for severe illness (and admitted to hospital and duration of symptoms < 10 days). We used the term severe illness to refer to this group of children. Children and caregivers were enrolled by dedicated surveillance nurses in the pediatrics ward or the outpatient clinic each week, based on the child’s symptoms fitting the case definition for the illness (mild or severe) and the parent’s willingness to consent. Following enrolment, a nasopharyngeal swab was collected for testing at the NICD by real-time polymerase chain reaction (rt-PCR) for RSV. Only data from children who tested positive for RSV were included in the costing analysis (i.e., RSV-associated illness).

### Data collection

Data for the costing study were collected by structured interview including caregiver out-of-pocket costs for the visit or admission at the time of hospitalization or clinic visit. These included the direct costs. such as transport, food, over-the-counter medication used, and cost of any private practitioner or traditional healer visits prior to study clinic visits or hospitalization. Indirect costs including loss of income were also collected from the caregiver. We applied the national minimum wage estimates to the days of lost income for the caregiver and to days of hospitalization for the unemployed caregiver. Minimum wage during the study period was $1.3 per day [6, 7].

To calculate healthcare provider costs (inpatient and outpatient) we used case-based data to calculate a patient day equivalent (PDE), which was multiplied by the number of days of care (number of days of admission for severe cases and 1 day for mild cases) to provide a case-cost to the healthcare system. All hospitalization and clinic costs were assigned to the healthcare costs as healthcare is free of charge to children aged < 5 years in South Africa. Itemized hospital (and clinic) costs and procedures (cost of facility services, administration, laundry, furniture, physician and nurse consultations, intensive care fees, chest X-rays, laboratory tests, and oxygen therapy) were obtained from the National Department of Health (NDoH) and the state price list of the National Health Laboratory Service (NHLS) [8, 9]. A similar exercise estimated outpatient-related healthcare system costs for mild cases. Direct costs for the caregiver (for mild and severe) were obtained during the structured interview in either the hospital or outpatient clinic. Direct costs included the cost of any pre-visit consultations with a healthcare provider (including doctors, traditional healers, and pharmacists), procedures (including X-rays), medication (prescribed or over the counter), and the cost of accessing healthcare (transport and food). Indirect costs of loss of income due to time is taken for all pre-visit and hospitalization time were collected.

For non-medically attended cases (mild and severe) costs outside of public facilities including over-the-counter (OTC) medications, consultation with traditional healers, direct non-medical costs (like transportation to collect medication), and indirect costs (loss of income) were obtained from the HUS (2013) in 4 of 9 provinces and adjusted for consumer price index [5].

To estimate the national economic burden of RSV-associated illness, 2013 to 2015 data were entered into a modified version of the World Health Organization tool for estimating the mean annual national cost burden, this included the healthcare system costs, caregiver costs, and non-medically illness) [4, 10]. We modified the model to include the direct and indirect cost estimates for caregivers for non-medically attended mild and severe illnesses as approximately 70% of mild and 50% of severe RSV-associated cases in children aged < 5 years are not medically attended in our setting [4].

We adjusted the total cost by the gross domestic product (GDP) deflator for South Africa from 2014 to 2022, and then by the annual average ZAR to USD exchange rate to compare costs collected to present costs [11].

Population data were obtained from Statistics South Africa as a mean annual population for 2011–2016 and case fatality ratios were obtained from surveillance data during the same time period [12]. We estimated the cost and cost burden of RSV-associated illness in 3-month age intervals in children aged < 1 year and as a single group for children aged 1–4 years.

We calculated years of life lost by applying the life expectancy estimates for South Africa in 2016 to the years lost for deaths in each age group [13].

## Results

### Costing study

We enrolled 527 children with mild illness and 675 children with severe illness in the costing study. The detection rate of RSV was 10% (57) in those with mild illness and 26% (178) in those with severe illness. Between 2% (children aged 9–11 months) and 4% (children aged 12–59 months) of children admitted with RSV-associated illness required intensive care admission (Table 1) and all of these were ventilated.

**Table 1:** The mean healthcare system costs per episode of respiratory syncytial virus (RSV)-associated mild and severe illness in children aged < 5 years, South Africa, 2014

Age group in months		0–2			3–5			6–8			9–11			12–59		
Item	Unit cost ($)	Proportion of children tested or requiring treatment	Duration of incurred cost (days)	Mean cost per episode ($)	Proportion of children requiring treatment	Duration of incurred cost (days)	Mean cost per episode ($)	Proportion of children requiring treatment	Duration of incurred cost (days)	Mean cost per episode ($)	Proportion of children requiring treatment	Duration of incurred cost (days)	Mean cost per episode ($)	Proportion of children requiring treatment	Duration of incurred cost (days)	Mean cost per episode ($)
**RSV-associated severe illness**																
Facility fee (24H)	84.20	1.00	6.24	525.11	1.00	4.74	399.37	1.00	4.37	367.66	1.00	5.60	471.50	1.00	4.13	347.61
Consultation (24H)	24.29	1.00	6.24	151.46	1.00	4.74	115.20	1.00	4.37	106.05	1.00	5.60	136.00	1.00	4.13	100.27
ICU (24H)	691.61	0.03	7.00	165.41	0.01	3.83	25.80	0.02	0.67	10.97	0.02	0.67	8.55	0.04	2.00	51.48
Chest X-ray	26.16	0.58	1.00	15.09	0.70	1.00	18.22	0.85	1.00	22.36	0.83	1.00	21.69	0.74	0.86	16.58
Oxygen (24H)	34.29	0.45	1.20	18.44	0.44	1.20	18.23	0.44	1.20	18.23	0.53	1.20	21.84	0.91	1.03	32.06
Antibiotic treatment	18.30	0.96	1.00	17.64	0.93	1.00	17.06	0.93	1.00	16.95	0.92	1.00	16.83	0.96	0.86	15.04
Other medications	8.04	1.00	1.00	8.04	1.00	1.00	8.04	1.00	1.00	8.04	1.00	1.00	8.04	1.00	0.86	6.89
HIV rapid	5.36	0.00	1.00	0.00	0.29	1.00	1.54	0.58	1.00	3.09	0.00	1.00	0.00	0.00	0.86	0.00
HIV ELISA	4.38	0.15	1.00	0.64	0.25	1.00	1.08	0.04	1.00	0.19	0.07	1.00	0.32	0.24	0.86	0.90
HIV PCR	30.54	0.28	1.00	8.43	0.23	1.00	7.02	0.44	1.00	13.58	0.48	1.00	14.76	0.31	0.86	8.03
HIV viral load	27.23	0.00	1.00	0.11	0.01	1.00	0.27	0.00	1.00	0.00	0.02	1.00	0.50	0.05	0.86	1.28
HIV CD4 +	14.55	0.00	1.00	0.03	0.01	1.00	0.09	0.00	1.00	0.00	0.00	1.00	0.00	0.04	0.86	0.47
Bacterial culture	4.11	0.89	1.00	3.65	0.68	1.00	2.80	0.83	1.00	3.40	0.69	1.00	2.85	0.59	0.86	2.09
Tuberculosis test	25.00	0.01	1.00	0.22	0.01	1.00	0.20	0.04	1.00	1.12	0.09	1.00	2.37	0.07	0.86	1.59
Blood cell count	4.64	0.58	1.00	2.71	0.57	1.00	2.66	1.00	1.00	4.64	0.83	1.00	3.87	0.24	0.86	0.96
CRP test	5.89	0.47	1.00	2.77	0.70	1.00	4.11	0.85	1.00	5.01	0.80	1.00	4.69	0.83	0.86	4.20
ESR test	2.77	0.07	1.00	0.19	0.13	1.00	0.37	0.18	1.00	0.51	0.32	1.00	0.87	0.17	0.86	0.41
Urea and creatinine	2.41	0.72	1.00	1.73	0.64	1.00	1.54	0.80	1.00	1.93	0.83	1.00	1.99	0.77	0.86	1.58
Cost per episode				921.66			623.59			583.71			716.68			591.44
Patient day equivalent (PDE)				147.70			131.56			133.57			127.98			143.26
**RSV-associated mild illness**																
Facility fee	8.4	1	1	8.4	1	1	8.4	1	1	8.4		1	8.4	1	1	8.4
Consultation	8.9	1	1	8.9	1	1	8.9	1	1	8.9		1	8.9	1	1	8.9
Antibiotic treatment	11.3	0.39	1	4.41	0.39	1	4.41	0.41	1	4.63	0. 42	1	4.75	0.45	1	5.09
Other medication	3.3	0.99	1	3.27	0.99	1	3.27	0.99	1	3.27	0.99	1	3.27	0.99	1	3.27
Total				24.98			24.98			25.20		1	25.29			25.66

The mean annual household income including government grants was $559 ($46.58 monthly) and the median was $475 (2.5–97.5% range $191–$1156) [14]. Children enrolled in the cost study did not differ with regard to demographic and clinical features as compared with children who were not enrolled in the costing study over the same period (data not shown).

### Healthcare system costs

Healthcare system costs were highest for the youngest infants (aged 0–2 months) at $921.66 per episode as compared with $623.59 for infants aged 3–5 months, $583.71 for infants aged 6–8 months, $716.68 for infants aged 9–11 months, and $591.44 for children aged 1–4 years. This cost is driven by a mean duration of hospitalization 6.2 days in infants aged 0–2 months as compared to 4.7 days, 4.4 days, 5.6 days, and 4.1 days in children aged 3–5 months, 6–8 months, 9–11 months, or 12–59 months, respectively. Healthcare system costs for mild illness were more uniform, ranging from $24.98 among infants aged 0–2 months to $25.66 in children aged 12–59 months per episode (Table 1).

### Cost to caregiver

For children with severe illness, the cost to caregivers was highest in the 0–2-month age group (total out-of-pocket and indirect costs) at $77.55 as compared to $62.21 for infants 3–5 months, $51.27 for infants 6–8 months, $72.19 for infants 9–11 months, and $66.77 for children. For children admitted with severe illness, the cost to the caregiver was between 110% ($51.27/$46.58) and 166% ($77.55/$46.58) of the mean monthly household income. Caregiver costs were similar across the age groups for children with mild illness (range $11.28–$11.99) (Table 2) and were an additional 26% of the mean monthly household income ($11.99/$46.58).

**Table 2:** Mean caregiver (out of pocket and indirect) costs per episode for medically-attended mild and severe illness in children aged < 5 years, South Africa, 2013–2015

Age in months	0–2	3–5	6–8	9–11	12–59
**Severe illness**					
Proportion of illness attended by caregiver (proportion of cases requiring caregiver in hospital)	0.94 (0.86–0.98)	0.89 (0.73–0.98)	0.96 (0.78–0.99)	0.833 (0.52–0.98)	0.68 (0.40–0.95)
Caregiver absenteeism (number of work days missed)	6.24 (4.34–8.06)	4.75 (3.33–6.18)	4.37 (3.06–5.68)	5.64 (3.95–7.33)	4.13 (2.89–5.37)
Out-of-pocket expenses caregiver/admission ($)	10.66 (1.81–19.48)	11.49 (3.41–19.57)	4.42 (0.31–8.53)	11.73 (1.07–22.39)	22.50 (4.47–45.97
Indirect cost caregiver/admission ($)	66.89 (46.82–86.96)	50.72 (35.50––65.94)	46.85 (32.80–60.91	60.46 (42.32–78.60)	44.27 (30.99–57.55)
**Mild illness**					
Proportion of illness attended by caregiver	1	1	1	1	1
Caregiver absenteeism (number of work days missed)	1	1	1	1	1
Out-of-pocket expenses caregiver ($)	0.57 (0–2.16)	0.57 (0–2.16)	1.28 (0–3.63)	0.71 (0–9.79)	0.86 (0.31–5.10)
Indirect cost caregiver/day ($)	10.71 (7.50–13.92)	10.71 (7.50–13.92)	10.71 (7.50–13.92)	10.71 (7.50–13.92)	10.71 (7.50–13.92)

### Cost burden

**Fig. 1:**
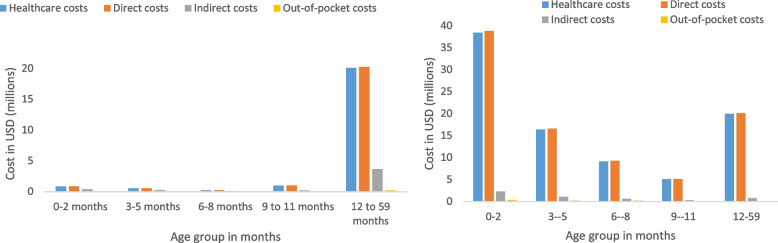
Mean annual national total cost burden of RSV-associated mild and severe illness in children aged < 5 years, South Africa, 2013 to 2015. **A** Mild illness. **B** Severe illness

The estimated mean annual cost of RSV-associated Illness in children aged < 5 years is $137,204.393 of which 76% ($111,742,713) are healthcare system incurred, 7% ($8,881,612) are out-of-pocket expenses and 17% ($24,225,801) are indirect costs. Thirty-three percent ($45,652.677) of the total costs were in the 0–2-month age group (Table 3, Fig. 1). The total costs in dollars adjusted for the GDP deflator and the ZAR dollar exchange rate from 2014 to 2021 were $132,991,552.97.

**Table 3:** Mean annual national cost burden of RSV-associated mild and severe illness in children aged < 5 years, South Africa, 2013 to 2015

	**Age group in months**					
Costs in USD ($)	0–2 *N* (% of total)	3–5 *N* (% of total)	6–8 *N* (% of total)	9–11 *N* (% of total)	12–59 *N* (% of total)	Total costs < 5 years
**Severe Illness (medically-attended)**						
** Total**	**41,082,860 (43)**	17,668,519 (19)	9,910,518 (10)	5,458,436 (6)	20,851,616 (22)	94,971,949
** Direct**	**38,801,193 (43)**	16,584,414 (18)	9,288,632 (10)	5,150,035 (6)	20,084,878 (22)	89,909,152
** Healthcare**	**38,435,544 (43)**	16,357,168 (18)	9,161,493 (10)	5,092,867 (6)	20,002,604 (22)	89,049,676
** Out of pocket**	**365,649 (43)**	227,246 (26)	127,139 (15)	57,168 (7)	82,274 (10)	859,476
** Indirect**	**2,281,667 (45)**	1,084,104 (21)	621,885 (12)	308,401 (6)	766,738 (15)	5,062,795
**Mild illness (medically-attended)**						
** Total**	1,262,599 (5)	830,645 (3)	347,148(1)	1,157,516 (4)	**23,918,961 (87)**	27,516,869
** Direct**	859,569 (4)	565,498(2)	244,517 (1)	980,019 (4)	**20,251,158 (88)**	22,900,761
** Healthcare**	841,433(4)	553,566 (2)	239,899 (1)	972,032 (4)	**20,086,107 (89)**	22,693,037
** Out of pocket**	18,136 (9)	11,932 (6)	4618 (2)	7987 (4)	**165,051 (79)**	207,724
** Indirect**	403,030 (9)	265,148 (6)	102,631 (2)	177,497 (4)	**3,667,802 (79)**	4,616,108
**Non-medically attended (mild and severe cases)**						
** Total**	3,307,218 (21)	1,546,779 (10)	1,546,779 (10)	601,610 (4)	**8,603,377 (55)**	15,605,763
** Direct**	1,057,580 (13)	510,314 (6)	510,314 (6)	291,669 (4)	**5,763,349 (71)**	8,133,226
** Indirect**	2,249,638 (30)	1,036,465 (14)	1,036,465(14)	309,941 (4)	**2,840,028 (38)**	7,472,537
**Total**						
** Total**	45,652,677 (33)	20,045,943 (15)	10,914,258 (8)	7,217,562 (5)	53,373,953 (39)	137,204,393
** Direct**	40,718,342 (34)	17,660,226 (15)	9,724,649 (8)	6,421,723 (5)	46,099,385 (38)	120,624,325
** Healthcare**	39,276,977 (35)	16,910,734 (15)	9,401,392 (8)	6,064,899 (5)	40,088,711 (36)	111,742,713
** Out of pocket**	1,441,365 (16)	749,492 (8)	323,257 (4)	356,824 (4)	6,010,674 (68)	8,881,612
** Indirect**	4,934,335 (30)	2,385,717 (14)	1,189,609 (7)	795,839 (5)	7,274,568 (44)	28,225,801
** Years of life lost**	17,812	10,431	5185	2684	3513	39,625

### Severe illness costs

The cost of RSV-associated severe illness was highest in the youngest infants (0–2 months), with 43% ($38,435,544/$89,049,676) of the healthcare system costs in this age group. Similarly, the highest proportions of the total out-of-pocket and indirect costs were in this age group; 43% ($365,649/$859,476) and 43% ($2,281,667/$5,062,795), respectively. Healthcare system costs for severe illness were relatively low in the 12–59 age group with 22% ($20,002,604/$89,049,676) of the healthcare costs in this age group (Table 3, Fig. 1).

### Mild illness costs

The largest cost burden of mild illness was in the 12–59-month age group, with 89% ($20,086,107/$22,693,037) of the healthcare system costs in this age group. Similarly, the cost burden in this age group for out-of-pocket costs and indirect costs are highest in this age group ($165,051 and $3,667,802, respectively) (Table 3, Fig. 1).

### Non-medically attended illness

Non-medically attended illness costs are higher in the older age group, similar to mild illness, with 55% ($8,603,377/$15,605,763) of costs in the 1–4-year age group. Non-medically attended illness accounted for 11% ($15,605,763/$137,204,393) of the total costs of RSV-associated illness.

### Years of life lost

We estimated years of life lost for deaths for children < 5 years with RSV-associated illness to be 39,625 years of which 45% (17,812) occurred in the 0–2-month age group (Table 3, Fig. 1).

## Discussion

In this analysis, we quantified the costs and cost-burden of RSV-associated illness, specifically the high costs and cost-burden of severe illness (hospitalized) in the youngest infants (aged 0–2 months), largely driven by high healthcare system costs in this age group. Costs are high throughout the first 6 months of life, the age groups targeted for new interventions. In contrast, for milder and non-medically attended illnesses, the majority of costs were in children aged 1–4 years, with significant costs of non-medically attended illnesses. This is the first analysis of the cost and cost burden of RSV-associated illness using pathogen-specific cost data derived from South Africa and provides important data to support cost-effectiveness models for interventions such as maternal vaccination and long-acting monoclonal antibodies aimed at youngest infants.

There are few data to compare to our analysis as few studies include finer age bands (most studies provide costs for children aged < 5 years) and most studies document the costs of pneumonia rather than pathogen-specific costs [15, 16]. Of the two studies conducted in South Africa, one describes the cost per episode of influenza-associated severe illness (all ages) as $685.60 and the second study reports the cost per episode as $1139 for all-cause pneumonia in children [14, 16]. In this analysis, we describe the average cost of RSV-associated severe illness for children aged < 5 years of $681.42. Our study describes a wide range of costs in the first 5 years of life and more especially in the first three months of life, allowing a more specific cost-effectiveness model for the age-specific interventions.

In a review article that included data from low and middle-income countries (LMIC) compared with high-income countries (HIC), the cost per episode of pneumonia ranged from $10–$1648 for inpatient care in LMIC (including data from South Africa), within the bounds of our estimate of $591.44–$921.66 for RSV-associated inpatient care. Similar to our study the costs for pneumonia were driven by the duration of hospitalization [15]. This review found a wide range of costs between different LMICs and differences in the management of hospitalized patients vs community care for pneumonia between countries, supporting the need for country-specific cost estimates to drive country-level decision-making.

Stenberg et al. updated the WHO-CHOICE models in 2018 with data from 30 countries to develop a robust model to estimate country-level costs [17]. Our average daily cost for hospitalization in the < 5-year age group ($136.81) was similar to estimates for Ecuador ($151.41) and Algeria ($131.41) and substantially more than estimates for Mozambique ($9.76). Although models like these are valuable, specifically for countries that do not have the resources to conduct costing studies, the extrapolations used may not reflect the actual costs in that setting. For example, exchange rate fluctuation and medical inflation may also vary between countries and over the time period. The level of care and the cost associated may also differ substantially within the country (for example urban vs rural areas or different provinces).

Healthcare is free to all children aged < 5 years in South Africa, which means that the South African government budget carries most of the burden of the costs of RSV-associated illness. The South African healthcare budget in 2016, was 38,563 billion Rand ($33.53 billion) [18]. Although the cost of RSV-associated illness is < 1% of the total healthcare budget, the cost saved by the introduction of a cost-effective intervention would release substantial funds to spend on strengthening other infant and child health prevention strategies [19]. When we adjusted the total cost by the GDP deflator and for the annual exchange rate (ZAR to USD), the cost update to 2021 was not much different from the 2014 costs, mainly due to the exchange rate increases at this time.

Although out-of-pocket and indirect costs are smaller proportions of the total costs of RSV-associated illness, the percentage of household income for these costs is high, specifically in a setting with high unemployment. In addition, the non-medically attended illness costs, specifically in the 1–4-year age group adds additional pressure on cash-strapped households, with these cost amounting to between 100 and 170% of the mean monthly household income.

Our study has several limitations. Although we enrolled children for a full year including the RSV season, there may be differences in severity and cost between years dependent on the subtype of RSV circulating. Our study included urban and rural sites but may not have been representative of all settings in the country. We were not able to follow cases enrolled into the HUS studies and so it is possible that children accessed care after the interview date leading to possible misclassification of non-medically attended cases. This is likely to be small as cases were asked about retrospective health-seeking behavior, in the past 14 days, i.e., most cases were not active at the time of the interview. The costs of long-term sequelae such as recurrent wheezing and loss of lung function following RSV infection are not captured in our study. In the South African Drakenstein cohort study a significant proportion of children experienced repeat admission and numerous follow-up visits to clinics for these complications [20]. The incidence rate ratio for RSV-associated recurrent wheezing requiring hospitalization following RSV-LRTI was 1.48 (95% CI 1.30–1.68), suggesting a significant burden and cost burden. We did not estimate the direct and indirect costs of RSV-associated deaths, for example, additional loss of income to the household associated with the death of a child and the costs of a funeral. Nor the societal costs associated with YLL. Our costing data were collected in a single year and may not reflect small changes in individual costs due to medical inflation or smaller fluctuations over the 3-year period.

## Conclusions

The highest costs to the healthcare system for RSV-associated pediatric illness in South Africa were in very young infants resulting in the highest cost burden in this age group; therefore, use of these higher per-illness cost estimates for interventions targeting young infants with either maternal vaccine or longer-acting mAb may result in higher quality cost-effectiveness analyses. The substantial cost of RSV-associated mild illness and non-medically attended illness in older children aged 1–4 years suggests early childhood vaccination against RSV may also be a cost-effective intervention.

## Data Availability

Data used for this manuscript is available through request to the first author, with approval from the surveillance program investigators.

## Abbreviations

$: United States dollar
BREC: Biomedical Research Ethics Committee
CI: Confidence interval
DHS: Demographic and health survey
GDP: Gross domestic product
HREC: Human Research Ethics Committee
HUS: Healthcare utilization survey
ILI: Influenza-like Illness
LMIC: Lower-middle-income country
LRTI: Lower respiratory tract infection
mAb: Monoclonal antibody
NDoH: National Department of Health
NHLS: National Health Laboratory Service
NICD: National Institute for Communicable Diseases
OTC: Over the counter
PCR: Polymerase chain reaction
PDE: Patient day equivalent
RSV: Respiratory syncytial virus
Rt-PCR: Real-time polymerase chain reaction
SARI: Severe acute respiratory illness
YLL: Years of life lost
ZAR: South African Rands
